# Population Perspectives on Nurturing Relational Health from Early Life: A Systematic Review Series

**DOI:** 10.1007/s10567-026-00565-7

**Published:** 2026-04-11

**Authors:** Craig A. Olsson, Jacqui A. Macdonald, Tracy Evans-Whipp, Alex Fischer, Primrose Letcher, Elizabeth A. Spry, Stephen R. Zubrick, the Australian Early Relational Health Network, Jacqueline Allen, Cath Chamberlain, Juli Coffin, Donna Cross, Alex Fischer, Jacinta Francis, Matthew Fuller-Tyszkiewicz, Rebecca Glauert, Melissa Green, Ross Homel, Kayla Mansour, Jennifer McIntosh, Shaun Mclaws, Siobhan M. O’Dean, Craig A. Olsson, Felicity Painter, Natasha Pearce, Naomi Priest, Lisa Ritland, Tim Slade, Sarah Whittle, Lucy Zhang

**Affiliations:** 1 Deakin Lifespan Institute (formerly SEED Centre for Lifespan Research), School of Psychology, Faculty of Health, Deakin University, Victoria, Australia; 2 Murdoch Children’s Research Institute, Centre for Adolescent Health, Department of Paediatrics, The University of Melbourne, Royal Children’s Hospital Campus, Victoria, Australia; 3 School of Cybernetics, Australian National University, Australian Capital Territory, Canberra, Australia; 4 Human Technology Institute, University of Technology Sydney, Sydney, Australia; 5 Kids Research Institute, The University of Western Australia, Perth, Australia; 6 Griffith University, Nathan, Australia; 7 The University of Melbourne, Melbourne, Australia; 8 Murdoch University, Murdoch, Australia; 9 University of Western Australia, Perth, Australia; 10 Deakin University, Victoria, Australia; 11 University of Technology Sydney, Sydney, Australia; 12 University of New South Wales, Kensington, Australia; 13 La Trobe University, Melbourne, Australia; 14 University of Sydney, Sydney, Australia; 15 Kids Research Institute, Perth, Australia; 16 Australian National University, Canberra, Australia; 17 University of British Columbia, Vancouver, Canada; 18 The University of Sydney, Sydney, Australia; 19 University of Melbourne, Melbourne, Australia

**Keywords:** Early relational health, Human development, Social networks, Bioecological framework, Population health

## Abstract

This paper provides the conceptual framework for a new review series that bring together the global literature on population approaches to nurturing relational health across the first three years of life. Early relational health is defined as ‘*the everyday interactions that happen between children and their carers across the many settings in which they live and grow … that meet universal needs for food and shelter, for safety, and for a sense of belonging, worth, identity and place that collectively establish secure foundations for lifespan health and development'*. The first four reviews describe (1) the relational ecology of early childhood, (2) associations with early brain development, (3) associations with relational outcomes beyond early childhood, and (4) evidence-based interventions for nurturing relational health within universal, population-based, settings (e.g., maternal-child health). The next four reviews tackle the deeper intergenerational question of what it means to raise children and young people in ways that also set developmental conditions for future roles as parents by describing (5) Australian Aboriginal ways of nurturing relationally healthy children and young people, (6) findings from intergenerational cohort studies, (7) population based interventions for nurturing relational health from childhood to parenthood (e.g., schools and communities), and (8) approaches to implementing evidence-based interventions in ways that ensure sustainability. An additional technical paper describes the development and testing of a new AI platform (LitQuest) that was purpose built to expedite title and abstract screening for the review series. Findings from the complete series are synthesised in a final paper that identifies critical gaps in the evidence base and provides recommendations for future research to advance population-level approaches to nurturing relational health from the earliest years of life.

## Introduction

We live in times of converging global emergencies, from vastly inequitable access to basic material needs (food, water, shelter), to rising geopolitical tensions and conflict, to the uncertain impact of generative AI and anthropogenic climate change which threatens the ecological equilibrium needed to support life as we know it (Zahidi, 2024). These share a common architecture: one built on injustice, pursuit of power over people, and the collapse of the most fragile threads of trust that connect us in our humanity and to our common environment.

This places relational health, between people, between nations, and with the environment, at the heart of our most pressing global challenges. This is true now; it has been true across all history; it will continue to be so into the future. Despite this, there remains a disquieting lack of dialogue around what it means to build a relationally well world; in particular, what it means to build relationally healthy families, schools, communities, as well as relationally healthy services, media, and government systems (Bronfenbrenner, 2005).

Contemporary society, it appears, has not yet prioritised relational health as a primary goal for building human and social capital, a situation akin to the lack of priority given to mental health until recently. This is despite living in a period of rapid social change, largely driven by technological advances, that is threatening to destabilise the connectivity we need to adapt and thrive. Symptoms are widespread, from an epidemic of loneliness, to apathy and disengagement, and to loss of trust in institutions and organisations, including democracy (Biddle et al., 2023).

Part of the difficulty in holding a discourse on relational health is its elusive nature. A significant challenge is that many aspects of relational life, and associated feeling states, have origins in the unrememberable but unforgettable experiences of the first years of life (Stern, 1985). But this may also be where an answer lies, because establishing a secure base from the beginning of life sets in place one of the important foundations of early development: the *capacity to trust in others* which sets in place a reciprocal process of human development that leads to relational wellbeing (Ainsworth, 1979; Erikson, 1964). It is difficult to underestimate the value of trust in others for all aspects of development; physical, cognitive, emotional, and moral. It is the most fundamental currency of relational health.

Trust emerges through the quality of interactions within the earliest and most formative of all relational experiences that children have with adult carers, particularly parents. Alongside relationships with other adults in microsystems such as childcare and community, these early relationships arguably form the most primary of all engines of human development. It is the way we evolved. Threats to early bonds introduce mistrust and vulnerability into development that can reverberate across the lifespan (Stern, 1985). To the contrary, secure early relationships build trust and create strong foundations for future healthy development (Bronfenbrenner, 2005).

A relationally secure start to life is also a fundamental human right and a developmental prerequisite for societal wellbeing (World Health Organisation, 2018). For most children, trust will emerge in abundance in social contexts defined by sensitive, attuned, and responsive care provision (McAnally et al., 2021), where parents have time to spend with their children; time to play, time to care, and time to support the early development of beliefs and values, consistent with family and cultural expectations. Through these most proximal relational processes of human development, children can become relationship-builders of the future, acquiring the skills needed to work together collectively, to compromise, to seek consensus and co-existence. These are the most fundamental of all skills needed to address the challenges of our time, and to ensure the survival and development of our species.

## Contexts of Relational Development

The contexts that prompt, facilitate or constrain early development are of central importance to building a relationally healthy world, and a collective capacity to address the global challenges of our time. In the most immediate context of the child’s family, parent mental health (Letcher et al., 2020; Spry et al., 2020), and parent relationship quality (Macdonald et al., 2022; Olsson et al., 2021), are critical determinants of a relationally secure start to life. Good parental mental health, and quality parent-parent relational bonds, contribute key emotional resources important to building strong bonds of trust between parent and child (Le Bas et al., 2020; O’Dea et al., 2023). These same assets can also ensure that parents have the capacity to meet the child’s needs in a timely manner.

However, for a substantial number of children, inconsistent, distracted, and anxious care provision will make trust more difficult to achieve from the beginning. Parent mental health difficulties, and parent-parent conflict can restrict the quality and quantity of the felt bond and the parental capacity to remain present and available to the infant (Brockington, 2011). For these children, life starts from an immeasurably more difficult position, where co-regulation of emotional states is tenuous, where trust in the availability of others is precarious, and where resultant patterns of adaptation create considerable risk for mental health and relational difficulties across the life course. This can be handed down across generations (Brockington, 2011).

It could be tempting to assume that within the scope of the first years of a child’s life, all relational influences in a child’s experience are contained in the context of the parent–child relationship. However, evidence from the developmental sciences confirms what parents and families have long known: that there are innumerable contexts that act upon parents and caregivers affecting their abilities to parent and influence the formation of trust and the quality of early relational health for them and their child (Booth et al., 2018; Bronfenbrenner, 1979, 2005).

Some of these contexts are determined by characteristics of the child, including genetic and other biological endowments that shape the nature of the bond between child and parent (Patton et al., 2018). For example, while an easy-going temperament is not essential to relational development, it can be helpful if present. However, other biologically based characteristics, such as prematurity, congenital disability, physical illness and neurodiverse capabilities, can place unique demands on parents. Such demands do not necessarily increase risk for bonding difficulties, but they have the potential to, at the very least, because of reduced time available for interaction and connection with the child.

Beyond the immediate child-parent family, extended family (grandparents, aunts/uncles, cousins) and wider social networks (friends, colleagues, significant others) play an important role in supporting and caring for parents too. This is often done through provision of instrumental, material and emotional supports that can replenish parental resources and support them to be physically and emotionally available to their offspring. To the contrary, the demands of dealing with, for example, pre-existing family relationship strain, together with new infant offspring needs, may strip parental resources and put relationships in both generations at risk (Infurna et al., 2020; Macdonald et al., 2023).

Institutional arrangements—well outside the immediate and extended family—further set the conditions of family life that shape opportunities for parents and offspring to build bonds of trust. Workplace conditions, childcare, schools, medical services, parent support groups, green spaces, and safe neighbourhoods collectively support parents and carers to raise emotionally secure offspring. To the contrary, non-family-friendly and otherwise toxic workplaces, lack of meeting places and green spaces, and resource-depleted or unsafe communities place additional burdens on parents as they seek to both care for their offspring and manage the challenges of their community contexts (Bronfenbrenner, 2005).

At the highest level, cultural norms and values, in the dominant culture or subcultures, can set powerful societal expectations for social behaviour, differentially concentrating resources across some families. Forms of social exclusion based on race, sex, or socioeconomic disadvantage, for example, continue to determine what is, and is not, possible within family life. They can become reified in institutional arrangements (courts, schools, churches, laws, regulatory frameworks) and are often slow to change in the face of more dynamic changes that occur and are adopted in the daily life of families. Norms and values form master narratives (McLean & Syed, 2015; Syed & McLean, 2022) that influence every other level of the social ecology of human development, akin to the ‘Outer Doll’ in a Russian Doll Set (Bronfenbrenner, 2005).

All nested ecologies of relational development change over chronological time too. From the earliest attachments in the first years of life, grows a foundation for forming increasingly sophisticated relationships throughout the rest of life. These onward relationships can take a variety of forms and transformations as represented in friendships, acquaintances, partners, marriages, memberships, agreements, contracts, and laws. Such relationships, and the actions arising from them, can support and strengthen social networks, norms and values and produce reciprocity and trust not just between individuals but also between groups, institutions, and indeed, between nations.

Attention must also be paid to intergenerational context, including the experiences of those who later become adult carers of children, well before they assume parenting roles and responsibilities. This much broader ‘ancestral’ frame of thinking has been central to Indigenous knowledge systems around the world, and across time. It acknowledges that material, human and social resources accrued in one generation can profoundly determine the next. For some, vulnerabilities accrued in prior generations will create shaky foundations (e.g., intergenerational trauma). For others, strengths accrued in prior generations (e.g., social and financial advantages) do the opposite. Either way, intergenerational cycles persist with force across generations. They cluster within families. They happen across multiple contexts. This makes breaking intergenerational cycles of risk difficult and resistant to ‘silver bullet’ approaches.

From an intergenerational *relational* perspective, for a bond of trust to develop between, for example, a parent and a child, the parent must have themselves developed sufficiently by the time of parenthood to be a foundation of integrity onto which the child may place its fragile and emerging trust. The connection between integrity and trust has long been recognised within psychodynamic theory; trust versus mistrust being the first conflict of early life, and integrity versus despair being the last in later life (Erikson, 1964). Integrity need not, however, be reserved as an accomplishment of later life. It could be fostered earlier, from childhood, to young adulthood and into parenthood, setting the foundations for trust in the next generation.

An intergenerational perspective requires a radical extension of thinking; one that pays attention to, and invests in, the years well prior to becoming a parent—the preconception years of a parent’s childhood, adolescence and young adult life. Few studies have sufficient longevity to examine the preconception years; however, those that do (Kretschmer, 2021; Olsson et al., 2020) are clearly showing that what parents bring to parenting is intricately linked to history, including mental health histories, experiences in earlier relationships (parents, peers, and partners), and material and educational resources (Thomson et al., 2021a, 2021b). This means that a complete understanding of the forces shaping early relational health must include history.

**Fig. 1:**
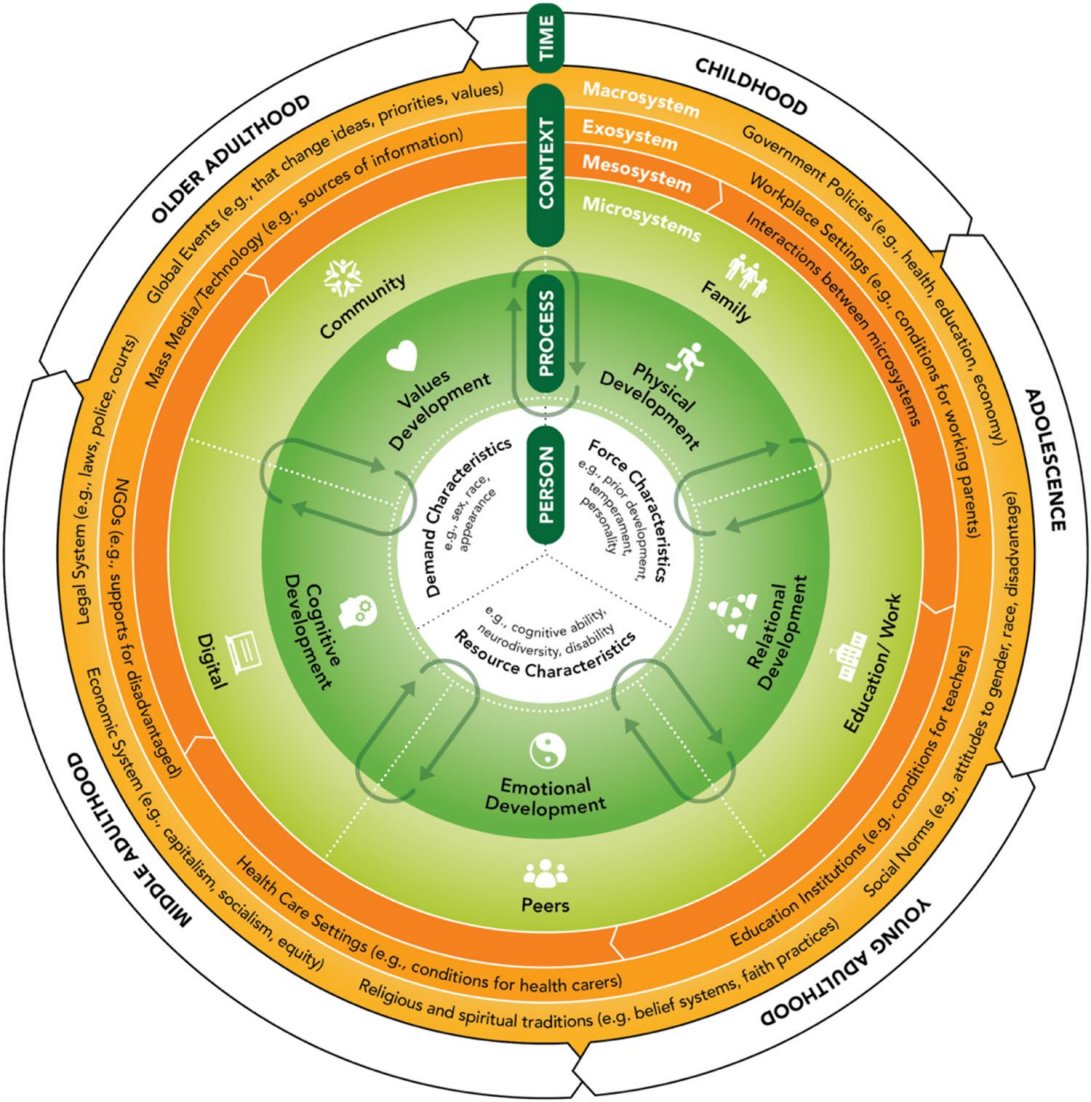
Bioecological model of human development

One of most eloquent conceptualisations of the social ecology of human development was first described more than half a century ago by Uri Bronfenbrenner (Bronfenbrenner, 1979, 2005)—see Fig. 1. He conceptualised five major systems. The first were the microsystems of family, early childhood care and community life. Connections between microsystems then formed a protective mesosystem, across which family, education and community settings provide mutually beneficial support in raising children well. These are embedded in an exosystem involving workplace, social media, and wider friendship networks, and macrosystems of common belief defined by culture or subculture. All systems change over time, changing in nature and aetiological significance across all ages and stages of the life course (chronosystem).

At the centre of this model are the daily reciprocal interactions that children have with people, objects, and symbols within the microsystems in which they live and grow. These interactions are considered the most *proximal processes of human development,* which within bioecological theory, places relational health as the singularly most important driver of development across the life course. Through relational processes, people interact with their contexts over time to acquire the skills and competencies needed to meet the demands of each age and stage of development. Within bioecological theory, this is referred to as the *Person-Process-Context-Time Model*, a model that places pre-eminence on the relationships that connect us to context, and in doing so, support our growth and development (Bronfenbrenner, 2004).

This interconnecting ecology of human relational development, from the earliest years of life, provides a powerful framework for conceptualising population level approaches to promoting child development through strengthening the relational contexts within which child development happens. It is a complex framework; one that recognises the need to invest broadly, and consistently over time, to achieve population wide gain in relational health. This special issue review series was inspired by a need to better understand these interacting systems in ways that inform population approaches to strengthening universal services systems.

## Nurturing Relational Health from Early Life Review Series

In 2022, a roundtable on ‘Early Relational Health’ was convened by the Paul Ramsay Foundation, Australia, to bring together experts on child development, from research, practice, government and non-government sectors, to commence a national dialogue on promoting relational health from early life (Paul Ramsay Foundation, 2023). The outcome was a joint commitment to a systematic review series describing ‘what we know’ and ‘what we don’t know’ about when, how and through what means we can best design public policy to nurture relational health from early life. We did not intend to review the clinical literature around repairing ruptured relationships. Our focus was on population approaches to nurturing relational health from early life, with a particular focus on universal approaches that could be applied to the whole population proportionate to need (*proportionate universalism*) (Jedan, 2021; Marmot & Bell, 2012). The aim of such an approach is to shift the population mean towards healthier relational states, and in so doing, improve overall population relational health, reducing demand for clinical intervention (Rose, 2001; Rose et al., 2009).

We defined early relational health for the review series as: *“The everyday interactions that happen between children and their carers across the many settings in which they live and grow – including parents, extended family and friendship networks, early education and health care providers, and the wider community—that meet universal needs for food and shelter, for safety (physical and psychological), and for a sense of belonging, worth, identity and place, that collectively establish secure foundations for lifespan health and development.”*

We conceptualised this series from an ecological perspective, recognising that bonds of trust established between parents and children, from the earliest years of life, are influenced by a constellation of relationships including those involving extended family, early childcare educators, other significant adult relationships in the community, the parent workplace, the economy, mass social media, and the norms and values embedded in national policy. An ecological approach acknowledges that promoting relational health from early life must consider all influences on the bond, both proximal and distal.

The dividends of investing in relational health from early life were further conceptualised from a life course perspective, one that extended beyond the formative years of development, and included historical forces that shape development well prior to conception. It is impossible to understand how to nurture relational health without a ‘big picture’ perspective on time: experiences from past shaping conditions of the present with implications for the future. And it is the future, with its array of global challenges, that most urgently requires secure relational foundations for us all.

Through this early relational health review series, we provide a comprehensive assessment and framing of what we know and what we need to learn if we are to ensure all children start life with strong relational foundations, break intergenerational cycles of trauma, and enable trustworthy social institutions to collectively adapt to multiple environmental, economic, technological and social changes. Our aim was to shift thinking from a ‘delivery state’, where services are understood in transactional ways, to a ‘relational state’, which recognises that human connections, not just services, drive outcomes (Mulgan, 2012; Plunkett, 2021).

To identify what is known about the nature, determinants and life course outcomes of patterns of early relational health (‘What Matters?’), we conducted systematic reviews (with narrative synthesis of study findings) of population based cohort studies with the aim of describing, (1) the relational ecology of child-caregiver relationships, (2) what we know about the short and longer-term (life course) consequences of early life relational strengths and difficulties and what new research needs to be undertaken to advance knowledge of longer term impacts, and (3) what we know about modifiable determinants of early life relational health, postnatally, antenatally, and intergenerationally, and what new research needs to be done to advance knowledge of modifiable determinants.

To identify evidence based interventions for nurturing relational health from early life (‘What Works?’) we conducted systematic reviews (with narrative synthesis of study findings) of community-based intervention studies with the aim of describing, (1) what we currently know about high quality evidence-based interventions designed to promote early life relational health, (2) what key gaps exist in evidence-based practice and what new research needs to be undertaken to address these gaps, and (3) how we maintain state-of-the-art knowledge of interventions, established and new, within the global literature.

To identify what is known about translating knowledge of what matters and what works into populations with the highest fidelity possible (‘What Translates?’), we concluded the series with two additional papers examining (1) how to bring together knowledge of what matters, with knowledge of what works, in ways that promote early life relational security, (2) how we achieve community-led translational initiatives that maximise the value of place-based knowledge as well as ensuring long term sustainability, and (3) what kinds of innovative study design approaches can be taken to evaluate the impact of translational activities on the quality of the early social and emotional environment.

To manage the large volume of research papers that needed to be screened for this review series (58,894 titles/abstracts), we developed an AI–based platform, LitQuest, that was designed to incorporate user decisions about paper eligibility (relevant/not-relevant) into an increasingly more accurate predictive model that ranks papers by relevance, incorporating a stop screening rule, to expedite title and abstract screening. Across the series, LitQuest saved time, human resources, and costs associated with the screening phase, reducing screening load by up to 78% across reviews (Fuller-Tyszkiewicz et al., 2025). LitQuest now provides a feasible basis for maintaining living reviews within the tight resource constraints typically available for this work.

## Review Series Structure

The complete set of reviews have been structured developmentally, commencing with reviews of the cohort and trials studies in early childhood, then extending intergenerationally to reviews of cohorts and trials that have focused on relational development across childhood, adolescence, young adulthood, and parenting transitions, which completes the life cycle back to our first set of reviews in early childhood.

Early childhood reviews start with a review led by Dr Siobhan O'Dean and team asking what is known about the social determinants of family life—in particular, extended social networks and systems that care for parents in ways that provide them with the time needed to build bonds of trust and support human development. This is followed by a second review led by Dr Felicity Painter and team examining the life course outcomes of bonds of trust that are established between parent and child from conception to three years of age. Aligned with this is a third review led by Dr Lucy Zhang and team examining longer term outcomes of early bonds on brain development. The final review in our early life set is by Professor Matthew Fuller-Tyszkiewicz and team and brings together all population approaches to promoting early relational health.

The series then pivots to a second set of reviews that tackles the deeper intergenerational question of what it means to raise children and young people in ways that also set developmental conditions for future roles as parents. In this we recognise the primary importance of raising children and young people well in its own right, without any consideration of next generation goals. However, for many people, parenting will be part of the life course, and so these experiences in early development will also set important foundations (both good and not so good) for the next generation.

Intergenerational reviews start with a review led by Australian Aboriginal researchers, Shannon McNeair, Professor Juli Coffin, Professor Cath Chamberlain and team, describing Australian Aboriginal led literature on relational knowledge systems for supporting a secure start to relational life, from childhood to parenthood. This is followed by a second review led by Associate Professor Jacqui Macdonald and team examining preconception predictors of next generation early relational health outcomes in long running intergenerational cohort studies. Completing the set is a third review led by Associate Professor Jacqui Allen and team that brings together all population level (public health) approaches to promoting relational health from childhood to parenthood.

A final review in this series by Dr Natasha Pearce and team examines evidence of effective translation into sustainable population service systems. The entire series is then brought together in a final synthesis led by Professor Steve Zubrick, with a particular focus on what we can do now to start strengthening social ecosystems to support early child development, and what further basic and applied research is needed going forward to strengthen the empirical rationale for investments across the full human ecosystem, with the aim of ‘making human beings human’.

In this review series we raise a challenge to think about nurturing a relationally secure start to life from a population perspective; one that brings more holistic frameworks to the table that capture the breadth of the social ecology around the developing child, that make better use of universal public health service systems to promote the relational health of micro- to macrosystems, and that more fully appreciate the intergenerational legacy that shapes the full social ecology of the developing human (Patton et al., 2018). In moving the conversation to include population approaches we build secure relational foundations for life for all.

## Data Availability

No datasets were generated or analysed during the current study.
